# Leaching of Phthalates from Medical Supplies and Their Implications for Exposure

**DOI:** 10.1021/acs.est.2c09182

**Published:** 2023-05-08

**Authors:** Wei Wang, Kurunthachalam Kannan

**Affiliations:** Wadsworth Center, New York State Department of Health, and Department of Environmental Health Sciences, School of Public Health, State University of New York at Albany, Empire State Plaza, P.O. Box 509, Albany, New York 12201-0509, United States

**Keywords:** phthalates, leaching, medical devices, intensive care unit, exposure

## Abstract

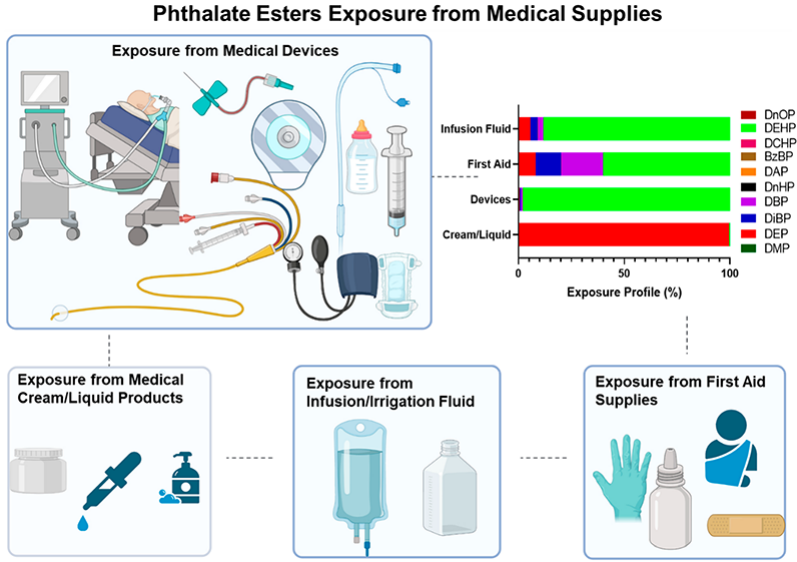

In this study, 72 single-use medical products, grouped into four categories, namely, creams/liquids (*n* = 8), medical devices (*n* = 46; 15 of 46 labeled “di(2-ethylhexyl)phthalate (DEHP)-free”), first aid products (*n* = 13), and intravenous (IV) infusion/irrigation fluids (*n* = 5), were collected from an intensive care unit in a hospital in New York State in 2015 and analyzed for the migration of 10 phthalates in ethanol/water (1:1) mixture for 1 h. The total phthalate concentration (Σ_phthalates_) leached from medical products ranged from 0.04 to 54,600 μg. DEHP was the major phthalate found in 99% of the samples analyzed, with the highest amount leached from respiratory support devices (median: 6560 μg). DEHP was also found at notable concentrations in products labeled as “DEHP-free”. Direct exposure to phthalates from the use of medical devices and first aid supplies and dermal intake from the use of creams/lotions were calculated. The highest DEHP exposure dose of 730 μg/kg bw/day was determined from the use of cannula for neonates. This is the first study to document the amount of phthalates leached from various medical supplies and associated exposures.

## Abstract 2

Respiratory support devices used in intensive care units in hospitals are a significant source of phthalate exposure in newborns.

## 1 Introduction

Phthalates, esters of *ortho*-phthalic acid, are widely used as plasticizers to improve the flexibility of polyvinyl chloride (PVC) plastics.^1^ Phthalates are present in consumer products, including personal care products (PCPs), pharmaceuticals, and medical devices.^2−6^ For over two decades, studies have shown that phthalates are reproductive and developmental toxicants and elicit carcinogenic, cardiotoxic, hepatotoxic, and nephrotoxic effects in laboratory animals.^7−10^ The effects of phthalates on the human endocrine system, especially on reproduction, are well documented.^11,12^ Di(2-ethylhexyl) phthalate (DEHP) and dibutyl phthalate (DBP) are reproductive toxicants.^13,14^ DEHP has been widely used as a plasticizer in medical devices.^6,15−17^ Nevertheless, it is not known whether other phthalate diesters such as diethyl phthalate (DEP)^18^ and butyl benzyl phthalate (BzBP) are used in medical devices.^19^

Because phthalates are not chemically bound to the plastic matrix, they can be released from single-use disposable medical devices, such as infusion, transfusion, and dialysis systems or feeding tubes, resulting in considerable exposure among patients in hospital settings.^17,20−22^ It is well established that medical procedures are a source of DEHP exposure for patients, particularly infants under intensive care, dialysis patients, and blood donors. The risk from DEHP exposure among neonates, infants, and pregnant women in intensive care units has been investigated by several authors.^23−25^ Leaching of DEHP from plastic indwelling medical devices used in the pediatric intensive care unit has been associated with impaired cognitive development in 4 year old children after critical illness.^15^ Despite this, the magnitude of exposure from each of the medical devices is not well known. Empirical data, although limited, have demonstrated a positive association between exposure and the use of PVC tubings, catheters, and gloves.^26^

Few earlier studies have reported the occurrence of phthalates in medical devices in the United States.^25,27^ Nevertheless, leaching/migration studies of a wide range of phthalates (in addition to DEHP) from medical supplies have not been conducted to date. Furthermore, quantitative assessment of phthalate exposure from each of the medical devices/products has not been performed, and such information is crucial to develop strategies to mitigate exposures. In this study, 72 medical devices and products collected in 2015 from an intensive care unit in a hospital in New York State were tested for the leaching of 10 phthalate diesters, with the aim of determining concentrations, profiles, and exposures.

## 2 Materials and Methods

### 2.1 Chemicals and Reagents

Ten phthalate diesters, namely, dimethyl phthalate (DMP), DEP, diisobutyl phthalate (DiBP), DBP, diallyl phthalate (DAP), di-*n*-hexyl phthalate (DnHP), benzyl butyl phthalate (BzBP), dicyclohexyl phthalate (DCHP), di(2-ethylhexyl) phthalate (DEHP), and di-*n*-octyl phthalate (DnOP), were analyzed. Nine d4 (deuterated) standards, d4-DMP, d4-DEP, d4-DiBP, d4-DBP, d4-DnHP, d4-BzBP, d4-DCHP, d4-DEHP, and d4-DnOP (AccuStandard, Inc. New Haven, CT), were used as internal standards. HPLC-grade ethanol was supplied by J. T. Baker (Phillipsburg, NJ). Ultrapure water (18.2 Ω) was generated using a Milli-Q system (Millipore, Billerica, MA). The chemical structures of phthalates analyzed in this study are shown in Table S1.

### 2.2 Sample Collection and Preparation

A total of 72 single-use medical devices and products comprising 8 cream/liquid samples, 46 devices, 13 first aid products, 4 intravenous (IV) fluids, and 1 irrigation fluid were obtained from an intensive care unit at a hospital in New York State in 2015. Description of the samples analyzed in this study is provided in Table S2. The medical supplies analyzed were categorized as medical devices, IV fluids/irrigation fluids, first aid products, and creams/liquids. Medical devices analyzed included baby products (e.g., nipple, pacifier, milk bottle, baby cloth, and baby diaper), medical tubing, syringe, catheter, connector, cannula, respiratory filter/support, IV fluid bag, blood pressure cuff and sensor pad, urine bag, plastic syringe, and clinical care supplies (e.g., fluidized positioner, lab diaper, and glove). In addition, first aid supplies (e.g., eye pad, gauze pad, abdominal pad, bandage, eyewash, burn cream, sterile alcohol prep pad, gauze, sting relief, plastic adhesive bandage, nitrile glove), medical creams/liquids (e.g., zinc oxide cream, liquid adhesive, baby wash), and IV fluids/irrigation fluids (saline, dextrose injection, sodium chloride injection, acetic acid injection, and fat emulsion IV infusion) were analyzed to represent varieties of medical supplies available in intensive care units. All samples analyzed were popular brands and distributed in hospitals throughout the United States. Samples were stored at 4 °C until analysis.

To assess leaching of phthalates from medical devices and products, an ethanol/water mixture (1:1, v/v) was used as the simulant for extraction.^22^ This mixture has been suggested as an extraction solvent/simulant to assess leaching of DEHP from medical devices.^22^ For medical devies (such as syringes, tubes, baby products, and clinical care products) and first aid products, samples were filled/immersed with the simulant and equilibrated for 15 min, which was followed by sonication for 1 h. This was based on the assumption that the maximum volume/contact areas of products were considered for exposure. Deuterated (d4) internal standards of DMP, DEP, DiBP, DBP, DnHP, BzBP, DCHP, DEHP, and DnOP were fortified in sample extracts prior to liquid–liquid extraction (LLE) with ethyl acetate/*n*-hexane (50:50, v/v), as described previously.^28^ The organic extracts were concentrated under a gentle stream of nitrogen to 2 mL, and an aliquot was transferred into a gas chromatograph (GC) vial for instrumental analysis. The sample extraction roughly simulated the clinical use conditions of the devices that come into contact with blood or other solutions (e.g., saline, nutrient solution, blood, or medication). For IV infusion and irrigation fluid samples, such as saline and injection solutions, LLE was performed directly, after transferring these fluids out of bags/bottles. The volume of fluid extracted varied, depending on the amount packaged, which ranged from 50 to 1000 mL. It was assumed that each package was used fully per application, once opened. For medical cream/liquid samples, ∼0.05 g (wet weight) was transferred and extracted twice by shaking with 4 mL aliquots of methyl *tert*-butyl ether in a 12 mL glass tube for 30 min (after spiking internal standards, 1 mL of Milli-Q water, and equilibration for 15 min), followed by centrifugation at 2000 × *g* for 20 min.^5^ The combined extracts were divided into two equal aliquots and concentrated under a gentle stream of nitrogen, and one aliquot was reconstituted in hexane for GC–MS analysis.

### 2.3 Instrumental Analysis

Identification and quantification of phthalate diesters was achieved using gas chromatography (Agilent Technologies 6890N) coupled with mass spectrometry (MS; Agilent Technologies 5973). A fused-silica capillary column (DB-5; 30 m × 0.25 mm i.d.; 0.25 μm film thickness) was used for chromatographic separation of analytes. The oven temperature was programmed from 80 °C (held for 1.0 min) to 180 °C at 12 °C/min (held for 1.0 min), increased to 230 °C at 6 °C/min, then to 270 °C at 8 °C/min (held for 2.0 min), and finally, to 300 °C at 30 °C/min (held for 12.0 min). The limit of quantification (LOQ) was calculated from the lowest concentration of the calibration curve and a nominal sample weight of 1.0 g. The LOQs ranged from 1 to 50 ng/g, depending on the analyte. Samples were diluted and reanalyzed when concentrations exceeded the calibration range of the instrument. The mass spectrometer was operated in the selected ion monitoring mode, and ion fragments at *m*/*z* 163, 279, and 149 were monitored for the quantification of DMP, DnOP, and 7 other phthalate diesters, respectively. The fragment ions at *m*/*z* 177 for DEP, 233 for DiBP and DBP, 223 and 206 for BzBP, 167 for DCHP, 167 and 279 for DEHP, and 279 for DnHP were monitored for the confirmation of target compounds. Ion fragment at *m*/*z* 167 was monitored for d4-DMP and *m*/*z* 153 was monitored for other internal standards. Further details of the analysis are provided in the Supporting Information and have been described in detail elsewhere.^3,4,29^

### 2.4 Quality Assurance and Quality Control and Data Analysis

Adequate precaution was taken to eliminate phthalate contamination that could arise from laboratory materials and solvents, during the analysis of samples. All glassware and GC vials were rinsed with acetone, hexane, and dichloromethane in sequence and baked in an oven at 500 °C overnight to remove any residual phthalates that may be present. For each batch of samples, two procedural blanks, a spiked blank, a pair of matrix-spiked samples, and duplicate samples were analyzed. Trace levels of DEP (9.5–16.5 ng), DiBP (34.8–69.9 ng), and DEHP (46.5–66.3 ng) were found in procedural blanks (*n* = 3) analyzed with medical devices, DEHP (15.3–33.4 ng) was found in procedural blanks (*n* = 2) of first aid samples, and DEP (n.d.–1.06 ng) and DEHP (43.1–65.2 ng) were found in procedural blanks of medical cream/liquid samples. The concentrations of phthalates in medical supplies were subtracted from the mean values found in procedural blanks. Instrumental calibration was verified by the injection of standards ranging in phthalate concentrations from 0.1 to 1000 ng/mL, and the regression coefficient (*R*) of all calibration curves was >0.99. The recoveries of target compounds spiked into medical devices (64–115%) and cream/liquid samples (90–106%) are shown in Table S1. The concentrations of phthalates in samples (both medical devices and cream/liquid) were calculated using an isotope-dilution method, based on the responses of corresponding deuterated internal standards (except for DAP, which was calculated based on the response of d4-DnHP). Duplicate analysis of randomly selected samples yielded a coefficient variation of <15% for the concentrations of target analytes. A midpoint calibration standard was injected after every 20 samples, as a check for instrumental drift in sensitivity, and a pure solvent (hexane) was injected as a check for carryover of target chemicals from sample to sample. Concentrations below the LOQ were assigned a value of zero for data analysis. Data analysis was performed using SPSS version 17.0 (Amsterdam, The Netherlands). Statistical significance was set at *p* < 0.05.

### 2.5 Calculation of Dermal Exposure Dose

On the basis of the median and maximum concentrations of phthalates measured in medical creams/liquids, we estimated daily dermal exposure doses (EDI_cream/liquid_) for adults, toddlers, infants, and neonates,^5^ as shown in eq 1
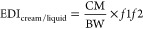
1where EDI is the daily dermal exposure dose (μg/kg bw/day), *C* is the measured concentration in creams/liquids (μg/g), *M* is the amount of daily use of creams/liquids (g), BW is the average body weight (kg), *f*1 is the retention factor (products retained by skin after use, *f* = 1),^30^ and *f*2 is the dermal absorption factor (*f*2 = 0.0011 for adults and *f*2 = 0.0021 for children).^5,31^ The values for *M* were obtained from the reports from the United States and Europe (1.4 g/day).^5,31^ The average body weights reported in the U.S. Exposure Factors Handbook for adults (80 kg), toddlers (14.6 kg), infants (8.3 kg), and neonates (5.35 kg) were applied.^32^

The daily exposure doses of phthalates through dermal absorption via baby clothes/diapers, blood pressure cuffs and sensor pads, and clinical care supplies (EDI_dermal_, μg/kg bw/day) were calculated using eq 2, where *D* is the amount of phthalate leached into the simulant in 60 min (μg). Each device/product was assumed to be used once a day.
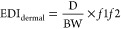
2

### 2.6 Calculation of Direct Exposure Dose

EDI_direct_ (μg/kg bw/day) was calculated for products that result in direct exposure from the use of medical devices (e.g., fluids that pass through the tubing). Each device/solution was assumed to be used once a day. An estimate of intake based on the amount leached into the simulant (ethanol and Milli-Q water) in 60 min was calculated using eq 3
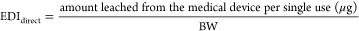
3

## 3 Results and Discussion

### 3.1 Phthalate Leaching from Medical Supplies

#### 3.1.1 Leaching from Medical Creams/Liquids

Medical creams/liquids are used in topical application for the treatment of wounds and rashes. Eight medical cream/liquid products (2 zinc oxide creams, 2 nasal zinc oxide creams, 2 baby washes, and 2 Mastisol liquid adhesives) were analyzed (see Table S2), and dermal exposure to phthalates was calculated (see Table S2). A few earlier studies reported the occurrence of phthalates in childcare lotion and cream products.^5,33^ Among 10 phthalates analyzed, the highest detection frequency was found for DEHP (100%) and DEP (75%), whereas DMP, DiBP, DBP, and BzBP were found only in a liquid adhesive (i.e., liquid band aid) sample. DEP was the major compound found at a mean concentration of 438 μg/g (range: n.d.–2050 μg/g), followed by DEHP (mean: 2.03 μg/g; range: 0.11–4.48 μg/g). A total phthalate (∑_phthalates_) concentration of 1750 μg/g was found in zinc oxide creams. Zinc oxide cream is a topical preparation that is applied to protect the skin from irritation, rashes, and sunburn.

#### 3.1.2 Leaching from Medical Devices

Medical devices are used in procedures such as blood transfusion, extracorporeal membrane oxygenation, parenteral infusion, and hemodialysis. Phthalates leached from these devices are a direct source of exposure in patients, especially among preterm neonates in intensive care units.^34^ The medical devices (*n* = 46; Table S2) investigated in this study were single-use products including those used in parenteral nutrition/drug administration, nasogastric intubation (plastic tube that is inserted through the nose, past the throat, and into the stomach), oxygen delivery systems, respiratory support and air filter, baby care and clinical care products (fluidized positioner/diaper/glove), and fluid drainage. Six of the 10 phthalates were found in >85% of the medical devices analyzed, suggesting widespread application of phthalates in medical products. DEHP was found in 98% of the devices with a median amount of 22.3 μg (mean: 2340 μg; range: n.d.–54,600 μg) leached into the simulant in 60 min. The highest amount of 54,600 μg DEHP was leached from a neonatal expiratory filter set. A few earlier studies^1,22,35^ reported migration of phthalates from medical tubings, blood bags, and syringes. DEHP is widely used in PVC, which is applied in disposable medical examination and surgical gloves, flexible tubings used in the administration of parenteral solutions, and hemodialysis treatment.^36^ DiBP was the second most abundant compound leached into the simulant at a median amount of 0.18 μg (range: n.d.–340 μg), followed by DBP (median: 0.14 μg; range: n.d.–19.7 μg). The highest amount of DiBP (340 μg) was leached from urine collectors for newborns. Among various medical devices tested, the amount of DEHP leached from the respiratory support and filter (*n* = 4, median: 6560 μg and maximum: 54,600 μg) was the highest. The median and maximum amounts of DEHP leached from other medical devices were as follows: clinical care supplies (fluidized positioners/diapers/gloves) (*n* = 4; median: 440 μg, max: 17,680 μg) > blood pressure cuffs/sensors (*n* = 4; 341 μg, 2620 μg) > urine bags (*n* = 1; 109 μg) > syringes (*n* = 1; 41.3 μg) > IV fluid bags (*n* = 4; 16.9 μg, 3260 μg) > nipples/pacifiers/milk bottles (*n* = 4; 4.16 μg, 47.9 μg) > catheters (*n* = 4; 5.11 μg, 30.5 μg) > baby clothes/diapers (*n* = 4; 4.16 μg, 47.9 μg) > cannulas (*n* = 5; 1.66 μg, 3910 μg) > tubings (*n* = 6; 1.31 μg, 1730 μg) > connectors/extensions (*n* = 5; 0.36 μg, 331 μg).

A high detection frequency (93%, *n* = 15) of DEHP in devices/products (in the general category of medical devices in this study) that were labeled “DEHP-free” was intriguing. However, the amounts of DEHP leached from those products were lower (median: 0.53 μg; range: n.d.–54,600 μg; *p* < 0.05) than those of other medical devices (median: 83.3 μg; 0.13–17,700 μg), suggesting the presence of smaller concentrations of DEHP in these products. The IV fluid bags were made of ethylene vinyl acetate (EVA) (labeled DEHP-free), PL 146 plastic (labeled as to contain DEHP), and PL325 plastic. Disposable syringes analyzed in this study were made of polypropylene resin. The highest amount of DEHP leached from IV fluid bags or syringes was from fluid bags made of PL146 plastic (3260 μg), followed by disposal plastic syringes (41.3 μg), whereas EVA and PL325 IV fluid bags leached 11.0–17.1 μg of DEHP into the simulant in 60 min. Although EVA bags were labeled as DEHP-free, >10 μg of DEHP was found to be leached into the simulant in 1 h.

#### 3.1.3 Phthalate Migration into IV Infusion Fluids

We investigated the migration of phthalates from IV fluid bags into four types of infusion fluids (pediatric parenteral nutrition, 600 mL; dextrose 5% injection, 50 mL; 0.9% sodium chloride, 250 mL; and fat emulsion 20% infusion) and one type of irrigation solution (0.25% acetic acid) stored in a sterile plastic bottle. The IV fluid bags were filled with the infusion fluids (instead of water–ethanol simulant), and the fluids were directly extracted with ethyl acetate/*n*-hexane. DiBP, DBP, and DEHP were detected in all solutions, with the median amount of DEHP at 165 ng, followed by DEP (10.4 ng), DiBP (6.76 ng), and DBP (4.65 ng). DEHP was found in infusion solutions contained in all IV bags including those labeled DEHP-free. Nevertheless, the amount of DEHP leached into IV fluid was 1–2 orders of magnitude lower than that leached into the water/ethanol simulant, for the same IV bag (median 16.9 μg; range 11.0–3260 μg). These results suggest that the type of fluid that passes through the medical device is an important determinant on the amount of phthalate leached. The highest amount of ∑_phthalates_ was detected in fat emulsion infusion (458 ng), which can be explained by the lipophilic nature of phthalates. The 0.25% acetic acid irrigating fluid leached the lowest amount of ∑_phthalates_ from the IV bags (177 ng). A combination of the chemical composition of IV infusion fluid and the type of IV fluid bag are major determinants of DEHP leached from these products. Furthermore, the surface area of the product that comes into contact with the solution can affect the amount leached. The average concentration of DEHP in IV infusion fluids was 1.55 μg/L, indicating that each liter of fluid contains 1.55 μg DEHP, which was lower than the value reported earlier for IV infusion fluid stored in the polyethylene terephthalate (PET) container (10 μg/L).^13^

#### 3.1.4 Leaching from First Aid Supplies

Leaching of phthalates was examined in 13 single-use first aid supplies including sterile eye pads, antiseptics, sterile abdominal pads, sterile gauze pads, bandages, sting relief (towelette), burn creams, plastic adhesive bandages, eyewash fluids, and nitrile gloves. The median amount of DEHP leached into the simulant from the first aid samples was 0.35 μg (range: 0.02–2.80 μg), which was followed by DBP (0.12 μg), DiBP (0.07 μg), and DEP (0.05 μg). The amount of DEHP leached from first aid supplies was considerably lower than that from medical devices (Table 1).

**Table 1:** Amounts of Phthalates Leached into the Water–Ethanol Simulant in 1 h from Medical Supplies and IV/Irrigation Fluids^a^

		DMP	DEP	DiBP	DBP	DAP	DnHP	BzBP	DCHP	DEHP	DnOP	Σ_phthalates_
cream/liquid unit: μg/g (*n* = 8)	mean	0.01	438	0.07	0.34	n.d.	n.d.	0.18	n.d.	2.03	n.d.	440
	median	n.d.	1.09	n.d.	n.d.	n.d.	n.d.	n.d.	n.d.	1.68	n.d.	5.81
	DF	13%	75%	25%	25%	0%	0%	13%	0%	100%	0%	100%
	range	n.d.–0.09	n.d.–2050	n.d.–0.28	n.d.–1.43	n.a.	n.a.	n.d.–1.44	n.a.	0.11–4.48	n.a.	0.23–2050
medical device unit: μg (*n* = 46)	mean	0.03	0.16	7.71	0.77	n.d.	0.02	0.08	n.d.	2340	n.d.	2350
	median	0.02	0.08	0.18	0.14	n.d.	n.d.	0.01	n.d.	22.3	n.d.	23.6
	DF	85%	96%	87%	98%	4%	15%	85%	0%	98%	7%	100%
	range	n.d.–0.09	n.d.–1.34	n.d.–340	n.d.–19.7	n.d.–0.03	n.d.–0.27	n.d.–0.66	n.a.	n.d.–54,600	n.d.–0.08	n.d.–54,600
first aid supply unit: μg (*n* = 13)	mean	0.01	0.14	0.23	1.17	n.d.	n.d.	0.05	n.d.	0.66	n.d.	2.26
	median	n.d.	0.05	0.07	0.12	n.d.	n.d.	n.d.	n.d.	0.35	n.d.	1.15
	DF	38%	100%	100%	92%	0%	0%	38%	0%	100%	0%	100%
	range	n.d.–0.06	0.01–0.68	n.d.–0.88	n.d.–13.4	n.a.	n.a.	n.d.–0.65	n.a.	0.02–2.80	n.a.	0.04–15.1
IV/irrigation fluid unit: ng (*n* = 5)	mean	2.91	18.3	12.6	16.6	n.d.	n.d.	0.17	n.d.	199	n.d.	249
	median	n.d.	10.4	6.76	4.65	n.d.	n.d.	n.d.	n.d.	165	n.d.	227
	DF	44%	78%	100%	100%	0%	0%	11%	0%	100%	0%	100%
	range	n.d.–9.77	n.d.–66.8	2.18–41.4	2.24–61.8	n.a.	n.a.	n.d.–1.49	n.a.	128–380	n.a.	150–458

a n.d.: not detected or below LOQ. n.a.: not available due to low detection frequency. DF: detection frequency.

### 3.2 Phthalate Profiles in Medical Supplies

**Figure 1:**
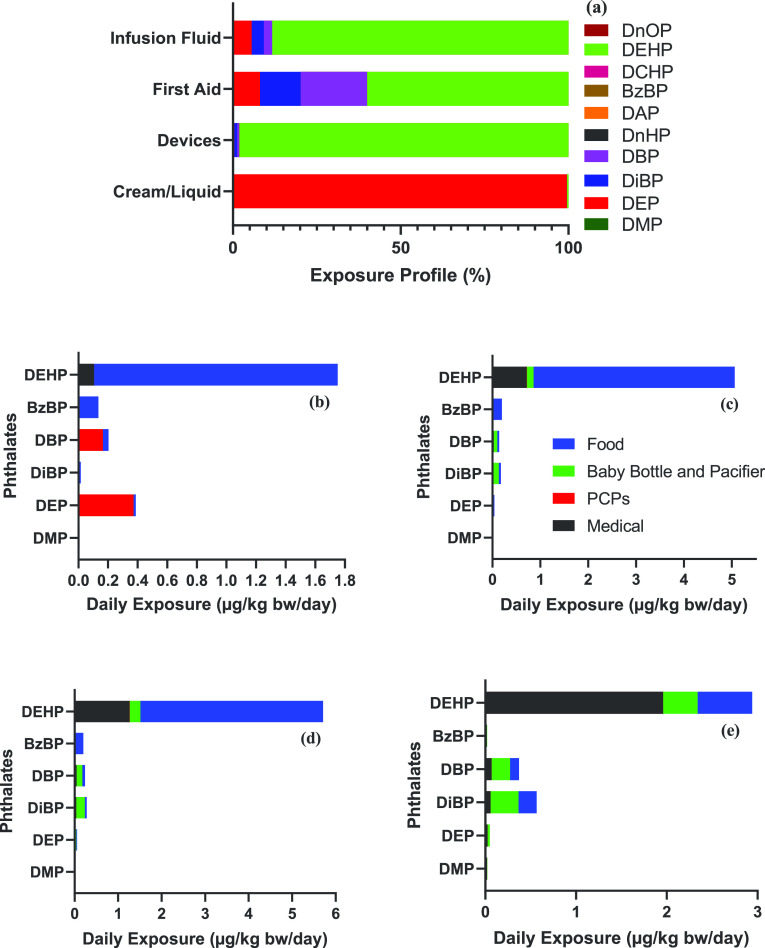
Distribution of phthalates in various medical products (a); contribution of different routes (food, PCPs, medical devices, and baby pacifiers/milk bottles) to the total daily exposure value (μg/kg bw/day) for adults (b), toddlers (c), infants (d), and neonates (e).

The patterns of exposure to phthalates varied among medical product categories. The overall phthalate exposures via medical supplies were dominated by DEHP, with an average contribution of 98%. This profile is consistent with those published earlier for various medical devices such as transfusion sets, plastic supplies for IV infusion, hemodialysis sets, dialysis bags, and tubings,^1,24,35,41^ even though different leaching and extraction methods were applied in those studies. Generally, leached DEHP predominated in medical samples made of PVC or LDPE. The proportion of DEHP, DEP, DiBP, and DBP found in IV fluids was 88, 5.6, 3.6, and 2.5%, respectively. DEP, DiBP, and DBP were also found in plastic package made of PET.^13^ Phthalate exposures via first aid products were mainly contributed by DEHP (60%), DBP (20%), and DiBP (12%). Considering the variety of materials used in first aid products, phthalates other than DEHP are expected to be present. Phthalate exposure from creams/liquids was dominated by DEP with an average contribution of 99.5% (Figure 1a); this profile is similar to that reported for PCPs.^5^ Our results confirm the pattern of phthalate application in medical cream/liquid products.

### 3.3 Human Exposure to Phthalates from Medical Supplies

The European Food Safety Authority (EFSA) has recommended tolerable daily intake (TDI) for DBP,^37^ BzBP,^38^ and DEHP^39^ at 10, 500, and 50 μg/kg bw/day, respectively.^40^ The U.S. Food and Drug Administration (FDA) suggested a TDI of 0.6 mg/kg bw/day for DEHP, with tolerable exposures of 42 mg/day for adults, 6 mg/day for children, and 2.1 mg/day for neonates (body masses of 70 kg for adults, 10 kg for children, and 3.5 kg for neonates).^22^

#### 3.3.1 Exposure via IV Fluids

Patients undergoing intensive therapeutic interventions can be exposed to higher levels of DEHP than the general population.^26^ A study reported that the maximum amount of DEHP exposure from blood bags was 0.7 mg/kg bw per use.^41^ In our study, the calculated median DEHP exposure dose for adults, toddlers, infants, and neonates from IV fluid infusions was 2.06, 11.3, 19.8, and 30.8 ng/kg bw per use event, respectively. The highest exposures were found for infants and neonates.^26,42,43^ Lower exposure doses calculated in our study can be attributed to the small median amount of phthalates leached into IV fluids in comparison to the earlier study that calculated based on blood stored in plastic bags for a long time.^41^ Thus, the duration of contact of fluids with plastic products and physicochemical properties of fluid can affect the amount leached into solution.

#### 3.3.2 Exposure via First Aid Supplies

Chemicals can enter through cuts, punctures, or scrapes into the blood stream. First aid supplies such as bandage can be a direct source of exposure to phthalates. The calculated exposure doses to DEHP (5–40 ng/kg/day for adults and 40–280 ng/kg/day for children) and DBP (2–190 ng/kg/day for adults and 10–1340 ng/kg/day for children) via the use of first aid products were below the TDI for both adults and children (body weight of 70 kg for adults and 10 kg for children were used).^22^ No earlier studies have reported phthalate exposure from first aid kits, and this study shows that such exposures are not negligible.

#### 3.3.3 Exposure via Medical Creams/Liquids

On the basis of the median and maximum concentrations of phthalates measured in creams/liquids, we estimated the daily exposure dose of these chemicals through dermal absorption for adults, toddlers, infants, and neonates.^5^ The highest dermal DEP exposure dose via medical creams/liquids for adults, toddlers, infants, and neonates was 30, 300, 520, and 810 ng/kg bw/day, respectively. The highest dermal DEHP exposure dose via medical creams/liquids was 0.06, 0.6, 1, and 2 ng/kg bw/day, for adults, toddlers, infants, and neonates, respectively. Exposure doses calculated from these products for newborns were considerably higher than those for adults. Messerlian et al.^44^ reported that aqueous gel used for obstetrical ultrasound in pregnancy can be a source of maternal and fetal exposure to phthalates. In utero exposure can occur, as phthalates cross the placental barrier and have been detected in the cord blood and amniotic fluid.

#### 3.3.4 Exposure via Medical Devices

Exposure doses to phthalates from the use of medical devices (including DEHP-free products) were calculated for adults, toddlers, infants, and neonates. Each device was assumed to be used once a day. The median exposure doses to DEHP from each medical device ranged from 0.001 to 0.96 μg/kg bw/day (Table 2), which were below the TDI value (0.6 mg/kg/day). Nevertheless, under maximum exposure scenarios (0.38–731 μg/kg bw/day), the doses exceeded the TDI value in certain cases, which suggests that the use of products containing high DEHP concentrations can pose health risks. The median EDIs of DEHP via nipples, baby pacifiers, and milk bottles were 0.14, 0.25, and 0.38 μg/kg bw/day, for toddlers, infants, and neonates, respectively. Among devices that contribute to direct exposure (e.g., catheters, cannulas, and tubings), the highest exposure to DEHP was from PVC-containing cannula, with the exposure dose calculated at 48.8, 267, 470, and 730 μg/kg bw/day for adults, toddlers, infants, and neonates, respectively. The DEHP exposure doses for neonates exceeded the TDI from the use of cannula.

**Table 2:** DEHP Exposure (μg/kg bw/day) Estimates via Different Routes from Medical Products Analyzed in This Study^a^

direct exposure							
		nipple/pacifier/milk bottle	tubing	catheter	connector/extension	cannula	total direct
median	adult	NC	0.02	0.06	0.005	0.02	0.11
	toddler	0.14	0.09	0.35	0.02	0.11	0.72
	infant	0.25	0.16	0.62	0.04	0.20	1.26
	newborn	0.38	0.25	0.96	0.07	0.31	1.96
maximum	adult	NC	21.6	0.38	4.14	48.8	74.9
	toddler	4.84	118	2.09	22.7	267	415
	infant	8.51	208	3.68	39.9	470	731
	newborn	13.2	323	5.70	61.9	730	1130

dermal exposure						
		baby cloth/diaper	cuff/sensor	clinical care supply	creams/liquids	total dermal
median	adult	NC	0.005	0.01	0.00002	0.01
	toddler	0.001	0.03	0.06	0.00024	0.11
	infant	0.001	0.05	0.11	0.00043	0.20
	newborn	0.002	0.07	0.17	0.00066	0.31
maximum	adult	NC	0.04	0.24	0.00006	0.28
	toddler	0.01	0.38	2.54	0.00064	2.93
	infant	0.01	0.66	4.47	0.00113	5.15
	newborn	0.02	1.03	6.94	0.00176	7.99

inhalation exposure		
		respiratory support
median	adult	0.08^b^
	toddler	0.45
	infant	0.79
	newborn	1.23
maximum	adult	0.68
	toddler	3.74
	infant	6.58
	newborn	10.2

a Not available/calculated.

b Inhalation exposure was estimated as the direct exposure from the amount leached into the products.

The total dermal DEHP exposure doses (sum of exposure from baby clothes/diapers, blood pressure cuffs/sensors, clinical care products [fluidized positioners/diapers/gloves] and creams/liquids) estimated for adults, toddlers, infants, and neonates were 0.01, 0.11, 0.20, and 0.31 μg/kg bw/day and 0.28, 2.93, 5.15, and 7.99 μg/kg bw/day, based on median and maximum amounts leached into the simulant, respectively. The sum of direct exposure doses from five categories of devices (nipples/pacifiers/milk bottles, tubings, catheters, connectors/extensions, and cannulas) analyzed in this study (Table 2, median: 0.11, 0.72, 1.26, and 1.96 μg/kg bw/day and maximum: 74.9, 415, 731, and 1130 μg/kg bw/day, for adults, toddlers, infants, and neonates, respectively) were lower than those previously reported for various medical products (5–8500 μg/kg bw/day for adults and 30–22,600 μg/kg bw/day for neonates)^45^ (Table S3). The differences can be attributed to the duration of contact, type/composition of the simulant, as well as the type of medical product tested. Furthermore, our exposure model for dermal contact did not account for the usage duration.

Assuming that the highest amount of DEHP was released into the air stream through respiratory supplies, DEHP exposure doses via the respiratory support, namely, nebulizers, therapy filters, transfer sets, and expiratory filters, were estimated at 0.02, 1.39, 11.7 and 54.6 mg/day, respectively, which were similar in the range of those reported by Roth et al.^46^ (0.017 to 100 mg/day). Hill^47^ reported EDI of DEHP (0.0004 to 0.001 mg/kg/day for adults) for patients undergoing respiratory therapy, based on measured concentrations in air stream. The median and maximum daily exposures were estimated to be 0.08, 0.45, 0.79, and 1.23 and 0.68, 3.74, 6.58, and 10.2 μg/kg bw/day for adults, toddlers, infants, and newborns, respectively.

The contribution of various sources [food, medical devices, nipples/baby pacifiers/milk bottles, and PCPs (including both rinse-off and leave-on)^5,48^] to the total intake of DEHP is shown in Figure 1b–e (only direct exposure pathways were considered for medical devices). The contribution of medical devices to total DEHP exposure increased in the following order: adults < toddlers < infants < neonates. The results suggest elevated DEHP exposure among neonates via medical supplies. The principal route of exposure to DEHP for adults is diet, with an estimated daily dose of 0.25 mg.^36^ PCPs were the major sources of DEP and DBP exposure for adults, whereas nipples/baby pacifiers/milk bottles were the major sources of DBP and DiBP exposure for infants and neonates.

The measured concentrations of DEHP in medical devices and IV fluids were lower than those reported in earlier studies (Table S3).^1,13,22,24,35,45,49−55^ The highest exposure doses to DEHP were reported from blood transfusions (using preserved blood in PVC blood bags) or hemodialysis.^45^ Different types of plastic materials have been reported to leach phthalates differently.^56^ For example, indwelling tubings can leach 21% of total DEHP content in 24 h of use, especially in lipophilic solutions such as parenteral solution or blood.^6,57^ The differences in concentrations measured in our study with those reported earlier may be related to the fact that we used more realistic migration and exposure scenarios, and tested different types of devices. Experimental conditions such as the type of simulants, surface area of products, and duration of contact can significantly influence the amount of phthalates leached. Furthermore, recent regulations on the use of phthalates in medical devices can be a factor in lower amounts found in our study. The high detection rates of DEHP in DEHP-free medical supplies may be a result of the sensitive analytical method used in our study. The median human exposure dose estimated via medical devices in this study was ∼157-fold higher for DEHP-containing products than DEHP-free products. However, this comparison was not based on the same type of medical devices. A median exposure dose of 0.53 μg/day via medical devices labeled DEHP-free is still of concern. Development of guidelines for an appropriate simulant and analytical method required for such assessments is needed. Although DBP, DEHP, and BBP were restricted in children’s toys at concentrations below 0.1%, regulations for phthalates in medical devices are needed.^58^

This is the first study to report exposure doses of phthalates via the use of various medical devices and supplies. However, the results should be interpreted with caution. We used an organic solvent-based simulant that may overestimate the amount of phthalates leached. Second, sample size was small for each type of medical device/product tested. Extrapolations were made for exposure calculations based on the migration levels in solutions. However, it should be noted that the occurrence of DEHP in medical devices (including those labeled DEHP-free) suggests the need for stringent regulations as these products constitute important sources of exposure in vulnerable populations, especially newborns.

In summary, we document migration profiles of phthalates from various medical supplies. DEHP was found to be leached from 99% of medical supplies tested, even in those that were labeled “DEHP-free”. Exposure doses to DEHP from IV infusion fluids, medical creams/liquids, and first aid supplies were <0.3 μg/kg bw/day, whereas those from medical devices were in the range of 0.005–730 μg/kg bw/day, with the highest exposure doses from cannulas for newborns. The highest amount of DEHP leached from the respiratory support ranged from 0.02 to 54.6 mg/day from a single use. The sum of direct exposure to DEHP from various medical products tested ranged from 74.9 to 1130 μg/kg bw/day for different age groups, while the sum of dermal exposure ranged from 0.28 to 7.99 μg/kg bw/day. Exposure doses of newborns to DEHP from medical devices exceeded the tolerance levels set by the FDA, under the maximum exposure scenarios. It should be noted that our exposure calculations from the devices assumed incidental and single-use scenarios, but frequent and long-term exposures can occur among patients. While the European Commission has set specific migration limits for plastic materials and articles in contact with food, no such limits have been set for medical devices. Efforts should be made to regulate phthalates in medical devices, and the safety of other alternative plasticizers that are currently used as replacements should be examined.^34^ The chemical safety of medical devices is of vital importance to help safeguard the health of patients, especially newborns, and efforts are needed to address the critical issue urgently.

## Supporting Information Available

Information regarding target analytes, samples analyzed, and previous studies describing phthalates in medical devices (PDF)

## References

[ref1] Gimeno P.; Thomas S.; Bousquet C.; Maggio A.; Civade C.; Brenier C.; Bonnet P. A. Identification and quantification of 14 phthalates and 5 non-phthalate plasticizers in PVC medical devices by GC-MS. J. Chromatogr. B: Anal. Technol. Biomed. Life Sci. 2014, 949–950, 99–108. 10.1016/j.jchromb.2013.12.037. 24480330

[ref2] Zhang B.; Zhang T.; Duan Y.; Zhao Z.; Huang X.; Bai X.; Xie L.; He Y.; Ouyang J.; Yang Y.; Wu Y.; Sun H. Human exposure to phthalate esters associated with e-waste dismantling: Exposure levels, sources and risk assessment. Environ. Int. 2019, 124, 1–9. 10.1016/j.envint.2018.12.035. 30639902

[ref3] Guo Y.; Zhang Z.; Liu L.; Li Y.; Ren N.; Kannan K. Occurrence and profiles of phthalates in foodstuffs from China and their implications for human exposure. J. Agric. Food Chem. 2012, 60, 6913–6919. 10.1021/jf3021128. 22703192

[ref4] Guo Y.; Kannan K. Challenges encountered in the analysis of phthalate esters in foodstuffs and other biological matrices. Anal. Bioanal. Chem. 2012, 404, 2539–2554. 10.1007/s00216-012-5999-2. 22535438

[ref5] Guo Y.; Kannan K. A survey of phthalates and parabens in personal care products from the United States and its implications for human exposure. Environ. Sci. Technol. 2013, 47, 14442–14449. 10.1021/es4042034. 24261694

[ref6] Vanhorebeek I.; Malarvannan G.; Güiza F.; Poma G.; Derese I.; Wouters P. J.; Joosten K.; Verbruggen S.; Jorens P. G.; Covaci A.; Van den Berghe G. Phasing out DEHP from plastic indwelling medical devices used for intensive care: Does it reduce the long-term attention deficit of critically ill children?. Environ. Int. 2022, 158, 106962 10.1016/j.envint.2021.106962. 34739923 PMC8685605

[ref7] Gray L. E.; Laskey J.; Ostby J. Chronic di-n-butyl phthalate exposure in rats reduces fertility and alters ovarian function during pregnancy in female long Evans hooded rats. Toxicol. Sci. 2006, 93, 189–195. 10.1093/toxsci/kfl035. 16763070

[ref8] Boberg J.; Metzdorff S.; Wortziger R.; Axelstad M.; Brokken L.; Vinggaard A. M.; Dalgaard M.; Nellemann C. Impact of diisobutyl phthalate and other PPAR agonists on steroidogenesis and plasma insulin and leptin levels in fetal rats. Toxicol 2008, 250, 75–81. 10.1016/j.tox.2008.05.020. 18602967

[ref9] Singh S.; Li S. S. Phthalates: Toxicogenomics and inferred human diseases. Genomics 2011, 97, 148–157. 10.1016/j.ygeno.2010.11.008. 21156202

[ref10] Ventrice P.; Ventrice D.; Russo E.; De Sarro G. Phthalates: European regulation, chemistry, pharmacokinetic and related toxicity. Environ. Toxicol. Pharmacol. 2013, 36, 88–96. 10.1016/j.etap.2013.03.014. 23603460

[ref11] Johnson K. J.; Heger N. E.; Boekelheide K. Of Mice and Men (and Rats): Phthalate-Induced Fetal Testis Endocrine Disruption Is Species-Dependent. Toxicol. Sci. 2012, 129, 235–248. 10.1093/toxsci/kfs206. 22700540 PMC3491958

[ref12] Li N.; Liu T.; Zhou L.; He J.; Ye L. Di-(2-ethylhcxyl) phthalate reduces progesterone levels and induces apoptosis of ovarian granulosa cell in adult female ICR mice. Environ. Toxicol. Pharmacol. 2012, 34, 869–875. 10.1016/j.etap.2012.08.013. 22986106

[ref13] Rastegari F.; Amin M.; Ebrahim K. Risk of Phthalate Exposure among Hospitalized Patient via Intravenous Fluids Receiving. Iran. J. Toxicol. 2017, 11, 33–38. 10.29252/arakmu.11.3.33.

[ref14] Wang Y.; Zhu H.; Kannan K. A review of biomonitoring of phthalate exposures. Toxics 2019, 7, 21 10.3390/toxics7020021. 30959800 PMC6630674

[ref15] Malarvannan G.; Onghena M.; Verstraete S.; van Puffelen E.; Jacobs A.; Vanhorebeek I.; Verbruggen S. C. A. T.; Joosten K. F. M.; Van den Berghe G.; Jorens P. G.; Covaci A. Phthalate and alternative plasticizers in indwelling medical devices in pediatric intensive care units. J. Hazard. Mater. 2019, 363, 64–72. 10.1016/j.jhazmat.2018.09.087. 30308366

[ref16] Panneel L.; Malarvannan G.; Jorens P. G.; Covaci A.; Mulder A. Plasticizers in the neonatal intensive care unit: A review on exposure sources and health hazards. Crit. Rev. Environ. Sci. Technol. 2022, 52, 3947–3972. 10.1080/10643389.2021.1970455.

[ref17] Bernard L.; Masse M.; Boeuf B.; Chennell P.; Decaudin B.; Durand N.; Genay S.; Lambert C.; Le Basle Y.; Moreau E.; Pinguet J.; Ponsonnaille V.; Richard D.; Saturnin N.; Storme L.; Sautou V. Medical devices used in NICU: The main source of plasticisers’ exposure of newborns. Sci. Total Environ. 2023, 858, 159994 10.1016/j.scitotenv.2022.159994. 36368381

[ref18] World Health Organization (WHO). Diethyl Phthalate. Concise International Chemical Assessment Document 52, Geneva, 2003.

[ref19] World Health Organization (WHO). Butyl Benzyl Phthalate. Concise International Chemical Assessment Document 17, Geneva, 1999.

[ref20] Chiellini F.; Ferri M.; Latini G. Physical-chemical assessment of di-(2-ethylhexyl)- phthalate leakage from poly (vinyl chloride) endotracheal tubes after application in high risk newborns. Int. J. Pharm. 2011, 409, 57–61. 10.1016/j.ijpharm.2011.02.024. 21356303

[ref21] Eckert E.; Münch F.; Göen T.; Purbojo A.; Müller J.; Cesnjevar R. Comparative study on the migration of di-2-ethylhexyl phthalate (DEHP) and tri-2-ethylhexyl trimellitate (TOTM) into blood from PVC tubing material of a heart-lung machine. Chemosphere 2016, 145, 10–16. 10.1016/j.chemosphere.2015.11.067. 26650574

[ref22] Luo H.; Sun G.; Shi Y.; Shen Y.; Xu K. Evaluation of the Di(2-ethylhexyl)phthalate released from polyvinyl chloride medical devices that contact blood. SpringerPlus 2014, 3, 58 10.1186/2193-1801-3-58. 24516786 PMC3916584

[ref23] Wahl H. G.; Hoffmann A.; Haring H.-U.; Liebich H. M. Identification of plasticizers in medical products by a combined direct thermodesorption-cooled injection system and gas chromatography-mass spectrometry. J. Chromatogr., A 1999, 847, 1–7. 10.1016/s0021-9673(99)00138-7. 10515691

[ref24] Kostić I. S.; Andjelkovic T.; Andjelkovic D.; Cvetković T. P.; Pavlović D. D. Determination of di(2-ethylhexyl) phthalate in plastic medical devices. Hem. Ind. 2016, 70, 159–164. 10.2298/hemind141129023k.

[ref25] Tickner J. A.; Schettler T.; Guidotti T.; McCally M.; Rossi M. Health risks posed by use of di-2-ethylhexyl phthalate (DEHP) in PVC medical devices: A critical review. Am. J. Ind. Med. 2001, 39, 100–111. 10.1002/1097-0274(200101)39:1<100::aid-ajim10>3.0.co;2-q. 11148020

[ref26] Green R.; Hauser R.; Calafat A. M.; Weuve J.; Schettler T.; Ringer S.; Huttner K.; Hu H. Use of di(2-ethylhexyl) phthalate–containing medical products and urinary levels of mono(2-ethylhexyl) phthalate in neonatal intensive care unit infants. Environ. Health Perspect. 2005, 113, 1222–1225. 10.1289/ehp.7932. 16140631 PMC1280405

[ref27] Calafat A. M.; Needham L. L.; Silva M. J.; Lambert G. Exposure to di-(2-ethylhexyl) phthalate among premature neonates in a neonatal intensive care unit. Pediatrics 2004, 113, 429–434. 10.1542/peds.113.5.e429. 15121985

[ref28] Onghena M.; Van Hoeck E.; Van Loco J.; Ibáñez M.; Cherta L.; Portolés T.; Pitarch E.; Hernandéz F.; Lemière F.; Covaci A. Identification of substances migrating from plastic baby bottles using a combination of low resolution and high resolution mass spectrometric analysers coupled to gas and liquid chromatography. J. Mass Spectrom. 2015, 50, 1234–1244. 10.1002/jms.3644. 26505768

[ref29] Tran T. M.; Kannan K. Occurrence of phthalate diesters in particulate and vapor phases in indoor air and implications for human exposure in Albany, New York, USA. Arch. Environ. Contam. Toxicol. 2015, 68, 489–499. 10.1007/s00244-015-0140-0. 25702083

[ref30] Wormuth M.; Scheringer M.; Hungerbuhler K. Linking the use of scented consumer products to consumer exposure to polycyclic musk fragrances. J. Ind. Ecol. 2008, 9, 237–258. 10.1162/1088198054084626.

[ref31] Wormuth M.; Scheringer M.; Vollenweider M.; Hungerbuhler K. What are the sources of exposure to eight frequently used phthalic acid esters in Europeans?. Risk Anal. 2006, 26, 803–824. 10.1111/j.1539-6924.2006.00770.x. 16834635

[ref32] U.S. Environmental Protection Agency. Exposure Factors Handbook. http://www.epa.gov/ncea/efh/pdfs/efh-chapter17.pdf (accessed March, 2013).

[ref33] Hubinger J. C. A survey of phthalate esters in consumer cosmetic products. J. Cosmet. Sci. 2010, 61, 457–465. 21241635

[ref34] Šimunović A.; Tomić S.; Kranjčec K. Medical devices as a source of phthalate exposure: a review of current knowledge and alternative solutions. Arh. Hig. Rada. Toksikol. 2022, 73, 179–190. 10.2478/aiht-2022-73-3639. 36226817 PMC9837533

[ref35] Veiga M.; Bohrer D.; Nascimento P. C.; Ramirez A. G.; Carvalho L. M.; Binotto R. Migration of phthalate-based plasticizers from PVC and non-PVC containers and medical devices. J. Braz. Chem. Soc. 2012, 23, 72 10.1590/s0103-50532012000100011.

[ref36] U. S. Department of Health & Human Services, Public Health Service, Agency for Toxic Substances and Disease Registry. Toxicological Profile for Diethyl Phthalate, 1993. 37647462

[ref37] Opinion of the scientific panel on food additives flavourings, processing aids and materials in contact with food (AFC) on a request from the commission related to di-butylphthalate (DBP) for use in food contact materials. EFSA 2005a, 242, 1–17.

[ref38] Opinion of the scientific panel on food additives, flavourings, processing aids and materials in contact with food (AFC) on a request from the commission related to butylbenzylphthalate (BBP) for use in food contact materials. EFSA 2005b, 241, 1–14.

[ref39] Opinion of the scientific panel on food additives, flavourings, processing aids and materials in contact with food (AFC) on a request from the commission related to bis(2-ethylhexyl)phthalate (DEHP) for use in food contact materials. EFSA 2005c, 243, 1–20.

[ref40] Cao X. L. Phthalate esters in foods: sources, occurrence, and analytical methods. Compr. Rev. Food Sci. Food Saf. 2010, 9, 21–43. 10.1111/j.1541-4337.2009.00093.x. 33467808

[ref41] Inoue K.; Kawaguchi M.; Yamanaka R.; Higuchi T.; Ito R.; Saito K.; Nakazawa H. Evaluation and analysis of exposure levels of di (2-ethylhexyl) phthalate from blood bags. Clin. Chim. Acta 2005, 358, 159–166. 10.1016/j.cccn.2005.02.019. 15893743

[ref42] Latini G. Potential hazards of exposure to di-(2- ethylhexyl)-phthalate in babies. Neonatology 2000, 78, 269–276. 10.1159/000014278. 11093005

[ref43] Hernández-Díaz S.; Su Y. C.; Mitchell A. A.; Kelley K. E.; Calafat A. M.; Hauser R. Medications as a potential source of exposure to phthalates among women of childbearing age. Reprod. Toxicol. 2013, 37, 1–5. 10.1016/j.reprotox.2013.01.001. 23333816 PMC3729282

[ref44] Messerlian C.; Mustieles V.; Wylie B. J.; Ford J. B.; Keller M.; Ye X.; Calafat A. M.; Williams P. L.; Hauser R. Ultrasound gel as an unrecognized source of exposure to phthalates and phenols among pregnant women undergoing routine scan. Int. J. Hyg. Environ. Health 2017, 220, 1285–1294. 10.1016/j.ijheh.2017.08.003. 28830670 PMC5671897

[ref45] Center for Devices and Radiological Health. Safety assessment of di(2-ethylhexyl)phthalate (DEHP) released from PVC medical devices; U.S. Food and Drug Administration: Bethesda, 2002.

[ref46] Roth B.; Herkenrath P.; Lehmann H. J.; Ohles H.-D.; Hömig H. J.; Benz-Bohm G.; Kreuder J.; Younossi-Hartenstein A. Di-(2-ethylhexyl)-phthalate as plasticizer in PVC respiratory tubing systems: indications of hazardous effects on pulmonary function in mechanically ventilated, preterm infants. Eur. J. Pediatr. 1988, 147, 41–46. 10.1007/bf00442609. 3422189

[ref47] Hill S. S. Analysis of contaminants in oxygen from PVC tubing in respiratory therapy, chromatographic components in electrochemical sensors, and a model for the degradation of electrical cable insulation. Ph.D. Thesis, University of Connecticut, 1997.

[ref48] Fromme H.; Gruber L.; Seckin E.; Raab U.; Zimmermann S.; Kiranoglu M.; Schlummer M.; Schwegler U.; Smolic S.; Völkel W. Phthalates and their metabolites in breast milk-Results from the Bavarian Monitoring of Breast Milk (BAMBI). Environ. Int. 2011, 37, 715–722. 10.1016/j.envint.2011.02.008. 21406311

[ref49] Mitani K.; Izushi F.; Kataoka H. Analysis of phthalate contamination in infusion solutions by automated on-line in-tube solid-phase microextraction coupled with high-performance liquid chromatography. J. Anal. Toxicol. 2004, 28, 575–580. 10.1093/jat/28.7.575. 15516316

[ref50] Gotardo M. A.; Monteiro M. Migration of diethylhexyl phthalate from PVC bags into intravenous cyclosporine solutions. J. Pharm. Biomed. Anal. 2005, 38, 709–713. 10.1016/j.jpba.2005.02.005. 15967299

[ref51] Khedr A. Optimized extraction method for LC–MS determination of bisphenol A, melamine and di(2-ethylhexyl) phthalate in selected soft drinks, syringes, and milk powder. J. Chromatogr. B: Anal. Technol. Biomed. Life Sci. 2013, 930, 98–103. 10.1016/j.jchromb.2013.04.040. 23727873

[ref52] Karle V. A.; Short B. L.; Martin G. R.; Bulas D. I.; Getson P. R.; Luban N. L.; O’Brien A. M.; Rubin R. J. Extracorporeal membrane oxygenation exposes infants to the plasticizer, di(2-ethylhexyl)phthalate. Crit. Care Med. 1997, 25, 696–703. 10.1097/00003246-199704000-00023. 9142038

[ref53] Loff S.; Kabs F.; Witt K.; Sartoris J.; Mandl B.; Niessen K. H.; Waag K. L. Polyvinylchloride infusion lines expose infants to large amounts of toxic plasticizers. J. Pediatr. Surg 2000, 35, 1775–1781. 10.1053/jpsu.2000.19249. 11101735

[ref54] U.S. Food and Drug Administration. Safety Assessment of Di (2-Ethylhexyl) Phthalate (DEHP) Released from PVC Medical Devices, Center for Devices and Radiological Health; U.S. Food and Drug Administration, 2001.

[ref55] Jaeger R. J.; Weiss A. L.; Brown K. Infusion of di-2- ethylhexylphthalate for neonates: a review of potential health risk. J. Infusion Nurs. 2005, 28, 54–60. 10.1097/00129804-200501000-00007. 15684905

[ref56] Gosetti F.; Bolfi B.; Robotti E.; Manfredi M.; Binotti M.; Ferrero F.; Bona G.; Marengo E. Study of endocrine disrupting compound release from different medical devices through an on-line SPE UHPLC-MS/MS method. Anal. Chim. Acta 2018, 1042, 141–154. 10.1016/j.aca.2018.07.028. 30428981

[ref57] Shang J.; Corriveau J.; Champoux-Jenane A.; Gagnon J.; Moss E.; Dumas P.; Gaudreau E.; Chevrier J.; Chalifour L. E. Recovery from a myocardial infarction is impaired in male C57bl/6 N mice acutely exposed to the bisphenols and phthalates that escape from medical devices used in cardiac surgery. Toxicol. Sci. 2019, 168, 78–94. 10.1093/toxsci/kfy276. 30398665

[ref58] Consumer Product Safety Improvement Act (CPSIA), Section 108, 2008. (available at http://www.cpsc.gov/cpsia.pdf) accessed Jan 24, 2023.

