# Supplementary material for: Leaching of Phthalates from Medical Supplies and Their Implications for Exposure
Source: Environ Sci Technol. 2023 May 8;57(20):7675–83. doi: 10.1021/acs.est.2c09182 (PMC10210534; doi:10.1021/acs.est.2c09182)
Supplement: Supplementary file 1 — es2c09182_si_001.pdf [file es2c09182_si_001.pdf]

Leaching of Phthalates from Medical Supplies and Their Implications for Exposure

Wei Wang[§] and Kurunthachalam Kannan[§,*]

[§]Wadsworth Center, New York State Department of Health, and Department of Environmental Health Sciences, School of Public Health, State University of New York at Albany, Empire State Plaza, P.O. Box 509, Albany, New York 12201-0509, United States

* Corresponding author at: Department of Pediatrics and Department of Environmental Medicine, New York University School of Medicine, New York, NY 10016. Tel: 212-263-1546, e-mail address: Kurunthachalam.Kannan@nyulangone.org (K. Kannan).

Number of Pages: 9 (including this page)

Number of Figures: 0

Number of Tables: 3

Instrumental Analyses

Ion fragments m/z 163, m/z 279, and m/z 149 were monitored for the quantification of DMP, DnOP, and seven other phthalate diesters, respectively. The fragment ions m/z 177 for DEP, m/z 233 for DiBP and DBP, m/z 223 and m/z 206 for BzBP, m/z 167 for DCHP, m/z 167 and m/z 279 for DEHP, and m/z 279 for DnHP were monitored for the confirmation of the target compounds. Ion fragment m/z 167 was monitored for d4-DMP and m/z 153 for other internal standards.

Table S1. Targeted Phthalates Information, Internal Standard, LOQ and Recovery Data.

Compound	Chemical Name	Formula	Exact Mass	Structure	Internal Standard	LOQ (ng/mL)	Mean Recovery%[a]	Mean Recovery%[b]
DMP	dimethyl phthalate	$C_{10}H_{10}O_4$	194	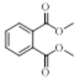	d4-DMP	5	114.92%	94.82%
DEP	diethyl phthalate	$C_{12}H_{14}O_4$	222	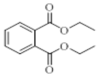	d4-DEP	2	98.83%	100.19%
DiBP	diisobutyl phthalate	$C_{16}H_{22}O_4$	278	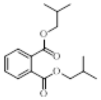	d4-DIBP	5	101.15%	99.10%
DBP	di-*n*-butyl phthalate	$C_{16}H_{22}O_4$	278	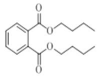	d4-DBP	5	100.82%	98.38%
DAP	diallyl Phthalate	$C_{14}H_{14}O_4$	246	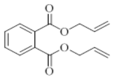	d4-DNHP	10	107.73%	94.23%
DnHP	di-*n*-hexyl phthalate	$C_{20}H_{30}O_4$	334	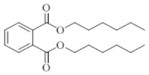	d4-DNHP	20	99.53%	97.09%
BzBP	benzyl butyl phthalate,	$C_{19}H_{20}O_4$	312	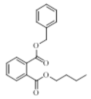	d4-BzBP	50	94.30%	93.23%

DCHP	dicyclohexyl phthalate	$C_{20}H_{26}O_4$	330	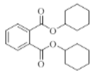	d4-DCHP	1	63.69%	90.25%
DEHP	di(2-ethylhexyl) phthalate	$C_{24}H_{38}O_4$	390	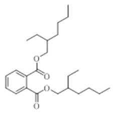	d4-DEHP	1	86.41%	100.11%
DnOP	di-*n*-octyl phthalate	$C_{24}H_{38}O_4$	390	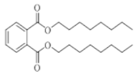	d4-DOP	50	70.22%	105.70%

[a] Recovery data for cream/liquid sample; [b] Recovery data for medical devices.

Table S2. Collected medical device samples and their product information.

	Material Label	Product	Note
**Medical Devices**			
	Cannula		
1	Contain PVC and DEHP	Nasal Cannula (infant)	
2	PHT, DEHP	BC2745 Infant Oxygen Therapy Nasal Cannula	
3	DEHP free	RAM Cannula	Newborn
4	DEHP free	RAM cannula ® Infant Nasal Cannula for Oxygen Administration	Infant
5	DEHP free	RAM cannula ® PREEMIE Nasal Cannula for Oxygen Administration	Preemie
	Tubing		
6	Contain PVC and DEHP	Uncuffed Tracheal Tube Murphy Eye, Oral/Nasal	
7	NA	Feeding Tube 0.5L 40cm-05Fr (PVC)	
8	Latex Free, NA	Parental Feeding Tube/Enteral feeding tube	
9	pHT, DEHP	Mallinckrodt® Oral/Nasal Tracheal Tube Cuffless	
10	NA	ANDERSEN® Nasogastric Tube	
11	PVC DEHP free	Nutrisafe 2 06Fr-L.50cm	
	Catheter		
12	PVC, DEHP free	Introcan Safety® PUR Catheter	
13	NA	BD Insyte™ Autoguard (Shielded I.V. Catheter)	
14	DEHP free	Argyle™ Polyurethane Umbilical Vessel Catheter	
15	DEHP free	Premicath	
	Connector/Extension		
16	DEHP free	MaxPlus Clear Needleless Connector	
17	DEHP free	MaxGuard T-connector Extension Set (microbore) with Injection Site (luer slip)	
18	DEHP free	MaxGuard Extension Set (microbore)	
19	DEHP	SmartSite Extension Set	
20	DEHP free	MaxGuard 4-way Stopcock	
	Cuff/Sensor		
21	NA	SoftCheck® Blood Pressure Cuffs	

22	NA	Neonatal/Adult Pulse Oximeter Adhesive Sensor	
23	NA	NEOTEMP® Neonatal Skin Temperature Sensor	
	IV Infusion/Irrigation Fluid Package		
	DEHP free	Pediatric Parenteral Nutrition Bag	
25	Contain PVC and DEHP	Dextrose5% Injection USP 50mL Bag	
26	VIAFLEX Plastic Container, NA	0.9% Sodium Chloride Injection USP 250mL Bag	
27	Plastic Pour Bottle, NA	0.25% Acetic Acid Injection, USP Bottle	
28	NA	Fat emusion 20% Infusion 2.6g Intravenous Package	
	Respiratory Transfer Set		
29	DEHP free	DAR® Neonatal D-X800 Expiratory Filter	
30	NA	Nonconductive Respiratory Therapy Filter	
31	NA	Respiratory Transfer Set	
32	NA	Airlife® Misty Max10^{TM} Nebulizer	
	Baby Product		
33	NA	Similac® Infant Nipple & Ring	
34	DEHP free	Soothier/Newborn pacifier	
35	NA	Breast Milk Storage Bottle	2 oz bottle
36	DEHP free	Soothier/Newborn Pacifier	
37	NA	Breast Milk Storage Bottle	2 oz bottle
38	NA	Baby Cloth	
39	NA	Baby Cloth	
40	NA	Baby Diaper	
	Clinical Care Products		
41	NA	Glove	
42	NA	Lab Diaper	
43	NA	Lab Diaper	
44	NA	Fluidized Positioners	
45	NA	Fluidized Positioners	
46	NA	U-Bag 24hour Urine Collectors Newborn	

IV I/Irrigation Fluid

1	NA	Pediatric Parenteral Nutrition	600mL
2	NA	Dextrose Injection USP 50mL	50mL
3	NA	0.9% Sodium Chloride Injection USP 250mL	250mL
4	NA	0.25% Acetic Acid injection, USP	1000mL
5	NA	Fat Emulsion 20% Infusion Intravenous	NA
**First Aid Products**			
1	NA	Eye Pad Sterile	
2	NA	Antiseptic	
3	NA	Combine ABD PAD Sterile	
4	NA	Gauze Pad Sterile	
5	NA	Bandage	
6	NA	Gauze	
7	NA	Gauze Pad Sterile	
8	NA	Sting Relief Insect Bite	
9	NA	Sterile Alcohol Prep Pad	
10	NA	First Aid & Burn Cream	
11	NA	Plastic Adhesive Bandage	
12	NA	Eye saline Eyewash (30mL)	
13	NA	Nitrile Glove	
**Cream/Liquid**			
1	NA	Curad Zinc Oxide Cream	
2	NA	Curad Zinc Oxide Cream	
3	NA	Nasal Zinc Oxide Cream	
4	NA	Nasal Zinc Oxide Cream	
5	NA	Baby Wash	
6	NA	Baby Wash	
7	NA	Mastisol Liquid Adhesive	
8	NA	Mastisol Liquid Adhesive	

Note: Medical supplies samples were categorized as medical devices, IV infusion fluid/irrigation fluid, first aid products, and cream/liquid.

Table S3. Studies Reporting Occurrence of and Exposure to Phthalates from Medical products/supplies.

Medical Devices (con. unit)	Material	Specification	Phthalates					References
			DMP	DEP	DBP	BzBP	DEHP	
Infusion solution (ng/mL)	PE, PP, EVA				6.7-58.9			Mitani et al.[49]
Infusion solution (mg/L)	PVC, PE						0.72-1.65	Veiga et al.[35]
Infusion solution (ng/mL)	PET	Ringer		4.6-16.4	2.7-9.1	nd-8.1	4.7-19.7	Rastegari et al.[13]
		Dextrose		nd-25.1	1.6-5.9	nd-2.1	6.3-18.8	
		Normal Saline		nd-12.1	1.8-4.7	nd-5.3	2.8-7.8	
		Injection Water		nd-24.1	nd-15.4	nd-7.3	nd-14.8	
Infusion solution (μg/mL)	PVC	0.9% NaCl					0.9-4.3	Gotardo and Monteiro[50]
Infusion solution (μg/mL)	PVC, LDPE	Dialysis solution, Saline, Ringer					3.58-21.16	Kostic et al.[24]
		Exposure					0.43 μg/kg/day[a]	
Respiratory tubing (μg/mL)	PVC	Condensate from water traps					<1-4100	Roth et al.[45]
		DEHP exposure					<0.7-4200 μg/h	
Medical devices contact blood	PVC	Transfusion set for single use (Blood Extraction)					0.068-0.073	Luo et al.[22]
DEHP released (mg)		Transfusion set for single use (Water Ethanol Mixture)					2.84	
		Leukocyte-reducing filter for single use (Water Ethanol Mixture)					1.2	
		Tube system for haemodialysis use (Water Ethanol Mixture)					11.6	
Medical devices (μg/g)	PVC	THF/EtOH extraction					>10000	Gimeno et al.[1]
Plastic material for intravenous infusion		PVC bag	nd	nd	nd	nd	115-170	Veiga et al.[35]
and from hemodialysis sets (mg/g)		EVA bag	nd	nd	nd	nd	2.86	
		Flexible tubing			nd-0.024		73.3-163	
		Rigid tubing			nd-0.06		17.8-23.7	
		Capillary	nd-0.03	nd-0.05	0.02-0.11	nd-0.09	4.75-11.3	
Medical Syringe 5mL, 20mL (μg/mL)							0.08-0.12	Khedr et al.[51]
		Exposure					0.61-1.61 μg/time	
Blood products (μg/mL)							1.8-83.2	Inoue et al.[41]
		Exposure					0.7 mg/kg/time	
Dialysis bag (mg/g)	PVC						23.0-325	Kostic et al.[24]

Tubing for dialysis (mg/g)	PVC				163-351	
Infusion bottle (mg/g)	LDPE				0.01-0.07	
Tubing from infusion set (mg/g)	LDPE				106-394	
CEMO circuit tubing (µg/g)					10.5-34.9	Karle et al.[52]
Neonates		Parenteral nutrition infusion exposure for neonates			5.1 mg/kg/day	Loff et al.[53]
Neonates		Total parenteral nutrition (TPN) administration exposure for neonates			2.5 mg/kg/day	FDA[54]
Neonates		Extracorporeal membrane oxygenation (ECMO) exposure for neonates			14 mg/kg/day	Jaeger et al.[55]
Various Medical Procedures Exposure (mg/kg/day)		Adults			0.005-8.5	CDRH[45]
		Neonates			0.03-22.6	
Median Exposure via Various medical Supplies (µg/kg/day)		Adults			0.005-0.06	This Study
(Dermal + Direct)		Toddlers			0.02-0.35	
		Infants			0.04-0.62	
		Neonates			0.07-0.96	
IV Fluid Infusion Exposure (ng/kg/day) (Direct)		Adults			2.06-4.75	This Study
		Toddlers			11.3-26.0	
		Infants			19.8-45.8	
		Neonates			30.8-71.1	
Respiratory Transfer Supplies (mg/day)					0.02-54.6	This Study
Cream/Liquid (ng/kg/day) (Dermal)		Adults	30		0.06	This Study
		Toddlers	300		0.6	
		Infants	520		1	
		Neonates	810		2	
First Aid Product Exposure (ng/kg/day) (Direct)		Adults		2-90	5-40	This Study
		Children		10-1340	40-280	

[a] The units for exposure data were indicated separately with the concentration unit.
